# Case Report: ProRoot MTA degradation in a compromised tooth: secondary trauma and acidic microenvironment leading to retreatment with Biodentine

**DOI:** 10.3389/fdmed.2025.1686600

**Published:** 2025-11-06

**Authors:** Saulius Drukteinis, Goda Bilvinaitė, Kerstin Galler

**Affiliations:** 1 Institute of Dentistry, Faculty of Medicine, Vilnius University, Vilnius, Lithuania; 2 Department of Applied Chemistry, Faculty of Chemistry and Geoscience, Vilnius University, Vilnius, Lithuania; 3 Department of Operative Dentistry and Periodontology, Friedrich-Alexander-University Erlangen-Nuernberg, Erlangen, Germany

**Keywords:** Biodentine, dental trauma, endodontic retreatment, mineral trioxide aggregate, orthodontic treatment, subluxation

## Abstract

**Background:**

Dental trauma can jeopardize the long-term success of previous endodontic treatments, especially in teeth affected by orthodontically induced inflammatory root resorption (OIIRR). This case presents the first documented association between secondary trauma-induced acidity and degradation of ProRoot mineral trioxide aggregate (MTA) in such compromised teeth.

**Case presentation:**

A 20-year-old male with a history of orthodontic treatment and severe apical root resorption sustained a subluxation injury to tooth 22, leading to pulp necrosis and apical pathosis. Initial endodontic management with ProRoot MTA achieved favorable outcomes at 6- and 18-month follow-ups. Two-and-a-half years later, secondary trauma occurred. Over the following 18 months, the tooth developed acute symptoms, a periapical lesion, and radiographic signs of MTA disintegration. Endodontic retreatment with Biodentine resolved the symptoms and achieved complete periapical healing, confirmed at 3, 6, and 18 months, and 4 years post-treatment.

**Conclusion:**

This case highlights MTA's susceptibility to acidic degradation in compromised conditions and supports Biodentine as a potentially more pH-resistant alternative. Clinicians should be vigilant when treating traumatized, orthodontically compromised teeth and prioritize restorative materials with high stability in hostile environments to minimize treatment failure risk.

## Introduction

Traumatic dental injuries (TDIs) are a significant clinical concern that can lead to severe functional and aesthetic problems (1). Their management strategies depend on factors such as the severity of injury, the extent of pulpal and periodontal damage, and the overall oral health of the patient (2). Timely diagnosis and appropriate treatment are crucial for ensuring the long-term survival of the affected tooth and preventing complications (3, 4).

Subluxation, which results in increased tooth mobility without displacement, is a common dental trauma, especially among children (2). While the immediate prognosis tends to be favourable, there is a risk of delayed complications, such as pulp necrosis and apical periodontitis (5). Regular monitoring and timely interventions are essential for achieving the best and more predictable outcomes.

Root resorption is a relatively common iatrogenic side-effect of orthodontic treatment. This condition, also known as orthodontically induced inflammatory root resorption (OIIRR), involves the loss of cementum and sometimes dentin at the root surface, potentially leading to shortened tooth roots (apical root resorption) (6, 7). Its prevalence varies according to factors such as treatment duration, the type and extent of orthodontic forces, and individual susceptibility (8). The occurrence of apical root resorption after orthodontic treatment can influence the prognosis of luxation injuries in different ways. Resorbed roots possess diminished structural integrity, rendering the tooth more susceptible to mobility and further resorption after TDIs (9). Therefore, an increased risk of tooth loss may be presented, especially in severe trauma cases (6).

Secondary dental trauma (SDT) refers to new injuries affecting teeth or their supporting structures that were already compromised by a previous traumatic incident (10). Re-injury can occur in up to 25% of previously traumatized teeth (11). SDT may lead to various complications, including periodontal issues, progressive external root resorption, alveolar bone loss, and new fractures, especially in teeth with structurally weakened roots (6, 8, 9).

While most research on traumatic dental injuries (TDIs) focuses on pulp and periodontal healing, there has been limited attention given to how SDT may affect the prognosis of previous endodontic treatments and the long-term integrity of root filling materials. To date, no published studies have directly linked SDT in a tooth compromised by irreversible pulpitis and OIIRR to the failure of hydraulic calcium silicate cements (HCSCs) such as ProRoot MTA. The mechanical strength and stability of MTA rely on an alkaline environment; however, acidic conditions, which are common in inflamed periapical tissues, can increase its solubility and compromise its structural stability (12–14).

This clinical case addresses this gap, presenting the dissolution of ProRoot MTA following SDT in a tooth previously treated due to primary trauma, and proposes a mechanism in which prolonged local acidity contributed to MTA degradation. The potential of Biodentine as a more acid-tolerant alternative is also discussed.

## Case presentation

This case report is described following the requirements of Preferred Reporting Items for Case Reports in Endodontics (PRICE), with key details outlined in Supplementary Figure S1. The patient gave written informed consent for all procedures and the publication of this report.

A healthy 20-year-old Caucasian man arrived at the dental clinic with the primary complaint of dental trauma that happened abroad 7 days ago after being attacked and beaten by a stranger. Immediately after the injury, the patient did not seek medical care. He took Ibuprofen to relieve moderate pain, which subsequently became mild and localized to the upper lip and incisors on the left. There was no loss of consciousness during the accident. After returning home, the patient contacted the dentist, who examined him and referred to the endodontist.

**Figure 1:**
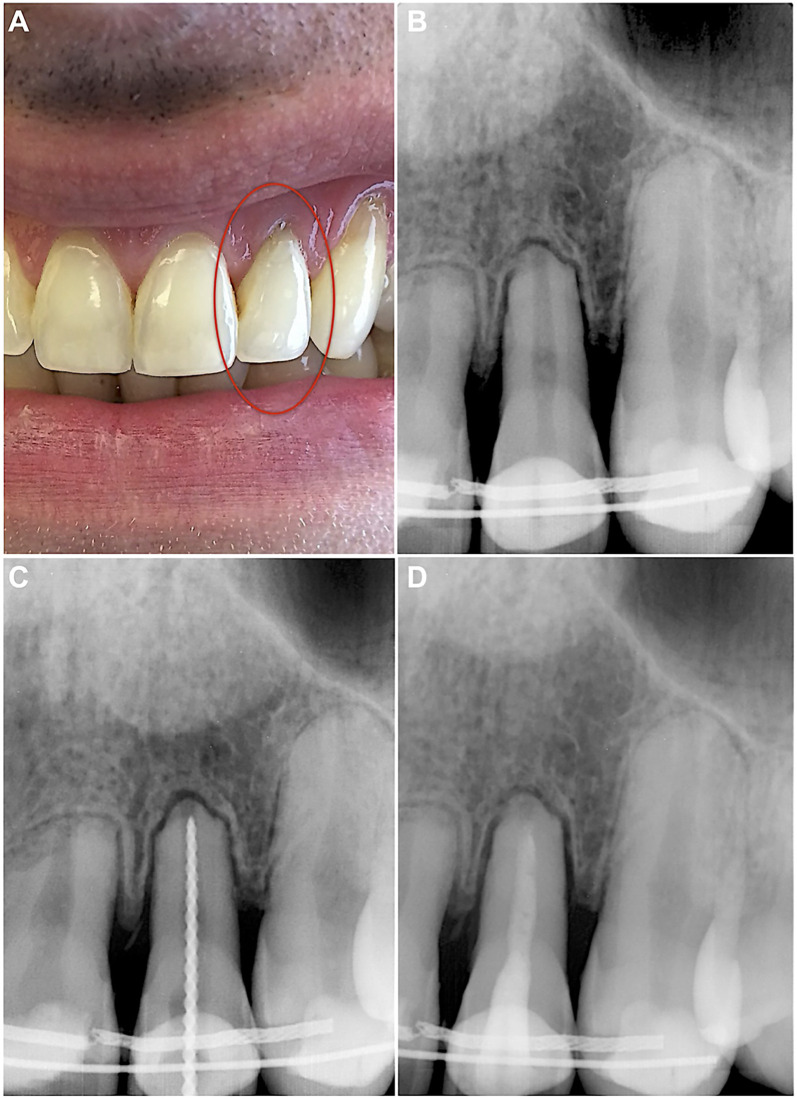
**(A)** Preoperative intraoral photo taken during the examination on the first visit. **(B)** Preoperative periapical radiograph revealing apical root resorption of teeth 21, 22, 23, and widened periodontal ligament of tooth 22. **(C)** Intraoperative periapical radiograph for WL determination with #45/0.02 K-File. **(D)** Postoperative periapical radiograph of cleaned and shaped root canal, filled with UltraCal XS paste. The access cavity was sealed with reinforced zinc oxide-eugenol restorative cement IRM.

During the anamnesis at endodontist, the patient indicated that bleeding from the gingival sulcus was visible at tooth 22 instantly after the injury. The tooth was painful to touch and bitting, and the sharp edge of the broken fixed wire retainer was felt. The patient underwent orthodontic treatment for 3 years and was currently in the retention phase for 4 months. Upon examination, no extraoral and intraoral changes were observed (Figure 1A). No tenderness to percussion was detected for teeth 21 and 23, and the sensitivity to cold and heat stimulus was normal. Meanwhile, tooth 22 was slightly tender to percussion, with a negative response to cold and heat tests. The periapical radiograph revealed the advanced apical root resorption of teeth 21–23 and a uniformly widened periodontal ligament of tooth 22 (Figure 1B).

After thorough anamnesis and clinical and radiological examination, the diagnosis of subluxation, along with pulp necrosis and apical periodontitis, was established for tooth 22. The endodontic treatment was initiated under local anaesthesia with 4% articaine and 1:100,000 epinephrine (Septanest, Septodont, Saint-Maur-des-Fosses, France). The procedure was carried out under a dental operating microscope (OPMI Pico; Carl Zeiss Meditec AG, Jena, Germany) which enabled inspection of the canal walls for cracks or fractures. The tooth was isolated with a rubber dam and Wedjets (Coltène/Whaledent, Langenau, Germany), and the crown was disinfected with an alcohol-based solution of 0.5% chlorhexidine. The endodontic access was prepared using high-speed Endo Access bur #1 (Dentsply Sirona, Ballaiques, Switzerland). The necrotic pulp was found in the pulp chamber and root canal.

The root canal working length (WL) was established using an apex locator and then confirmed radiographically (Figure 1C). The canal was shaped using hand files and the “apical box” technique up to #80/0.02 K-File (Dentsply Sirona, Ballaiques, Switzerland). After each instrument, the root canal was irrigated with 5 ml 3% sodium hypochlorite (NaOCl; Ultradent Products Inc., South Jordan, UT, USA) using disposable syringes and 29-G NaviTip needles (Ultradent Products Inc., South Jordan, UT, USA). At the end of shaping, a 5 ml 17% EDTA solution (Ultradent Products Inc., South Jordan, UT, USA) was used. The canal was then dried with sterile paper points and filled with calcium hydroxide paste UltraCal XS (Ultradent Products Inc., South Jordan, UT, USA) (Figure 1D). The endodontic access was sealed with intermediate restorative material IRM (Dentsply Sirona, Ballaiques, Switzerland). Finally, the broken retainer was fixed using light-cured resin-bonded composite Charisma (Kulzer GmbH, Hanau, Germany).

**Figure 2:**
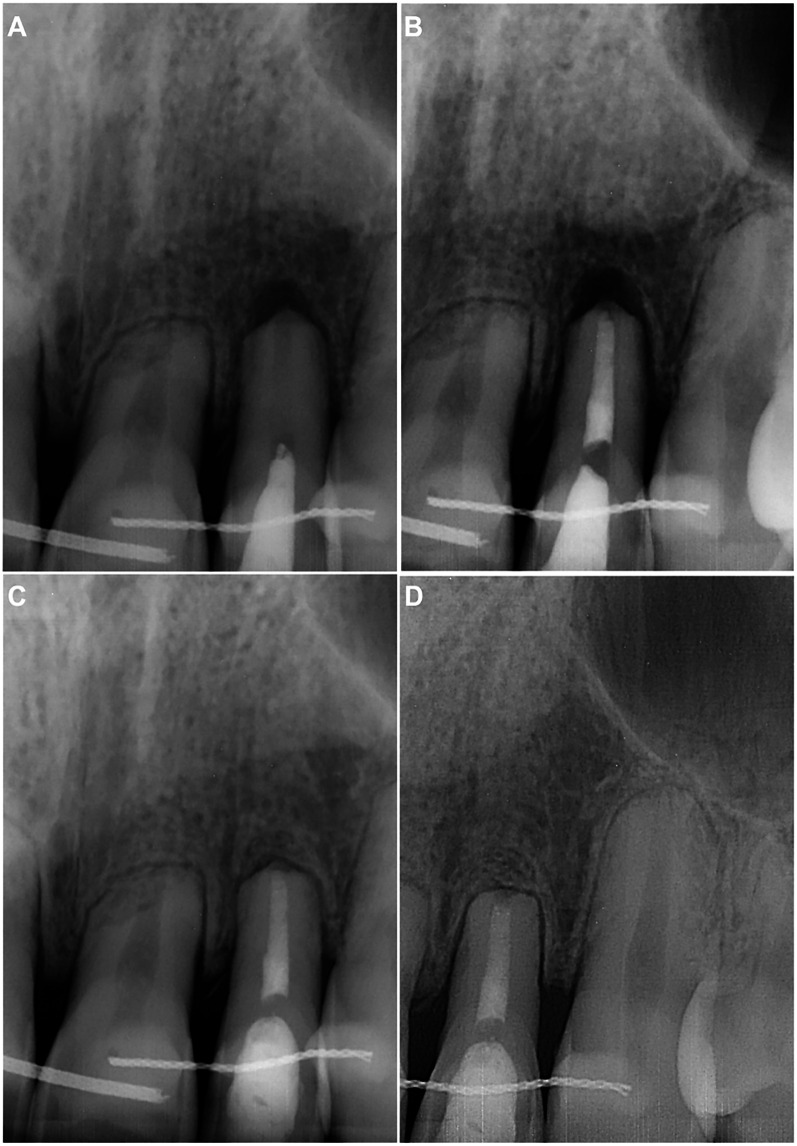
**(A)** Preoperative periapical radiograph demonstrating the empty root canal and periapical lesion of tooth 22. **(B)** Postoperative periapical radiograph after the root canal filling with ProRoot MTA White, followed by a wet cotton pellet and temporary restorative material IRM in the endodontic access cavity. **(C)** Six-month follow-up periapical radiograph showing a completely healed periapical lesion on tooth 22. **(D)** Eighteen-month follow-up periapical radiograph demonstrating no pathological changes in the tooth and periapical tissues.

Although the second visit was scheduled after 2 weeks, the patient returned 4 weeks later due to his illness. The patient had no complaints, and no clinical signs were found for teeth 21–23 on examination. The periapical radiograph showed that the calcium hydroxide paste was washed out, and there was a periapical lesion on tooth 22 (Figure 2A). After the aforementioned anaesthesia protocol, isolation, and disinfection, the temporary filling was removed. The procedure was performed under a dental operating microscope, which confirmed the absence of visible cracks or fractures. The root canal was irrigated with 5 ml 3% NaOCl, 5 ml 17% EDTA, and 5 ml sterile physiological saline and then dried with sterile paper points. No additional shaping was performed at this stage, as the canal had already been enlarged during the initial instrumentation, and further preparation risked compromising the structurally weakened root. Due to the apical root resorption and the resulting direct communication with periapical tissues, the canal was filled with ProRoot MTA White (Dentsply Sirona Inc, Charlotte, NC). The material was mixed according to the manufacturer's instructions and condensed to a level 2 mm below the canal orifice. A moist sterile cotton pellet was placed, the access cavity was isolated with IRM, and the radiograph was taken to assess the quality of obturation (Figure 2B). After 48 h, the IRM and cotton pellet were removed, and fully set MTA was covered with a thin layer of glass ionomer cement Fuji IX (GC CORPORATION, Tokyo, Japan). The crown was restored with light-cured composite Charisma.

The patient returned without complaints for scheduled follow-ups at 6 months (Figure 2C) and 18 months (Figure 2D). No clinical or radiological signs of endodontic pathology were found on examination. The periapical lesion of tooth 22 was completely healed. The next follow-up was scheduled after a year.

**Figure 3:**
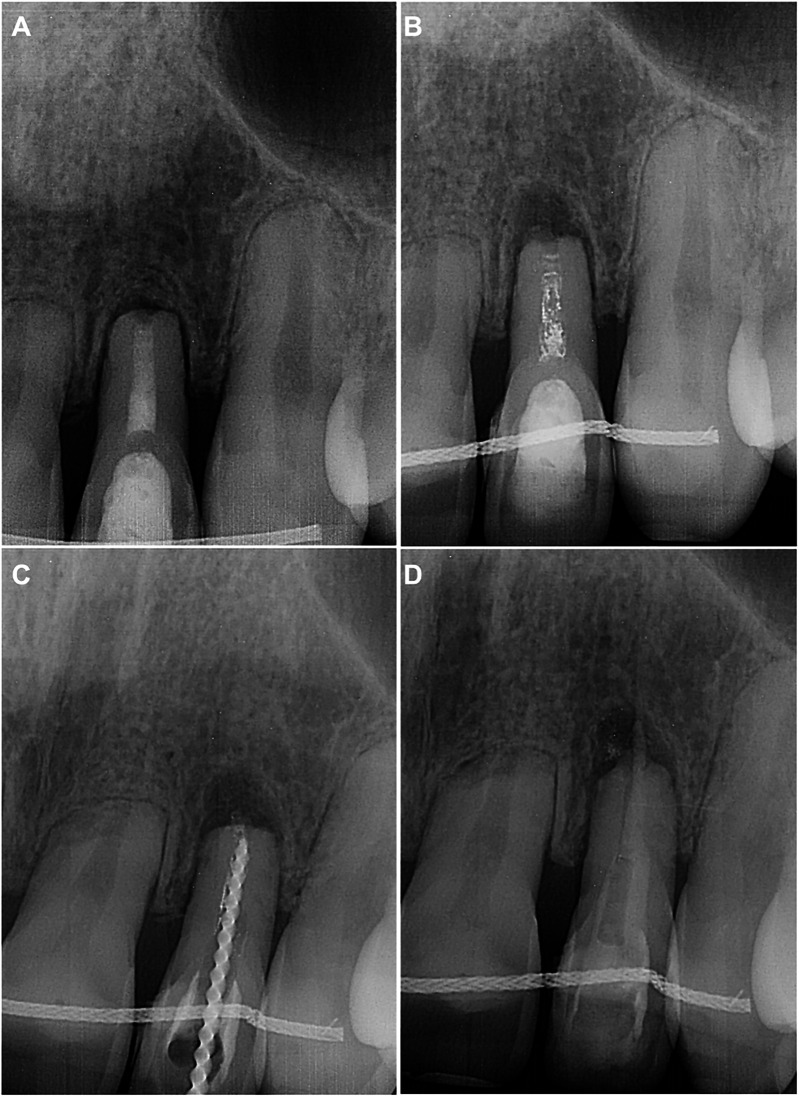
**(A)** Periapical radiograph performed during examination 2 weeks after the secondary dental trauma. **(B)** Eighteen-month follow-up periapical radiograph demonstrating changes in homogeneity of the root canal filling, along with a periapical lesion on tooth 22. **(C)** Intraoperative periapical radiograph for retreatment WL determination. **(D)** Intraoperative periapical radiograph after placing Biodentine in the apical and middle thirds of the root canal.

The patient returned for examination 14 months later, reporting that he accidentally fell down and re-injured his upper lip and front teeth 2 weeks ago. After the accident, the patient took Ibuprofen for 2 days to manage the pain. The patient had no complaints other than mild discomfort in tooth 22. Upon examination, no signs of endodontic pathology were observed. The retention wire was intact, and tooth 22 was slightly tender to percussion. The periapical radiograph revealed a uniformly and slightly widened periodontal ligament (Figure 3A). A follow-up was scheduled 1 month later to monitor the situation.

The patient did not adhere to the follow-up protocol and returned 18 months later, reporting spontaneous, throbbing pain and sensitivity on touching tooth 22. Upon examination, no extraoral or intraoral changes were observed. However, the tooth 22 was acutely tender to percussion. The periapical radiograph revealed a periapical lesion, along with a noticeable disintegration and decreased homogeneity of root canal filling (Figure 3B). Endodontic retreatment of the tooth was immediately initiated by removing the coronal restoration under the aforementioned protocol of local anaesthesia, isolation and disinfection. The procedure was performed under a dental operating microscope, which again revealed no signs of cracks or fractures. The dissolved MTA, discovered in the canal, was easily removed with hand K-files and the WL was regained (Figure 3C). The root canal was then reshaped up to #90/0.02 K-file, following the same irrigation protocol of NaOCl and EDTA as described above. The canal was filled with UltraCal XS paste, and the temporary filling IRM was placed.

The patient had no complaints after 2 weeks, and no clinical signs were found for tooth 22. Under local anaesthesia, the temporary crown seal was removed from the isolated and disinfected tooth. The irrigated (5 ml 3% NaOCl, 5 ml 17% EDTA, 5 ml sterile physiological saline) and dried canal was then filled with Biodentine (Septodont, Saint-Maur-des-Fosses, France), mixed according to manufacturer's instructions (Figure 3D). After the initial setting, the tooth was restored with light-cured resin-bonded composite Brilliant (Coltene-Whaledent, Allstetten, Switzerland).

**Figure 4:**
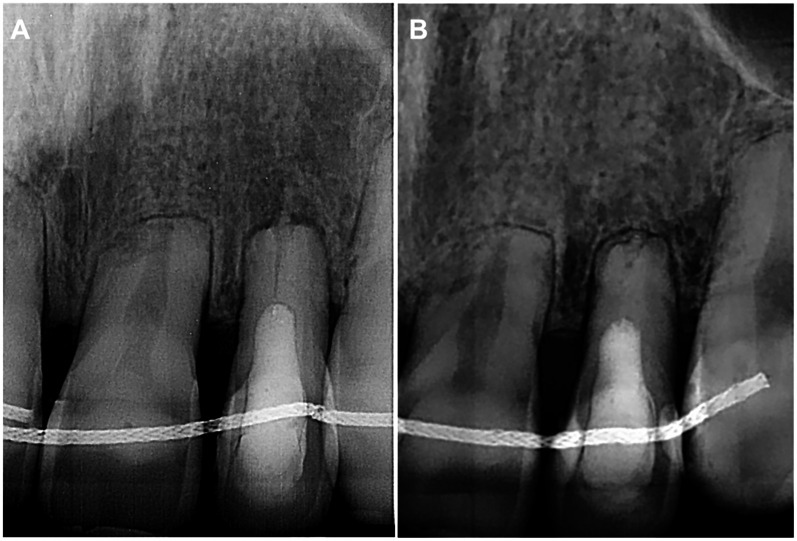
**(A)** Three-month follow-up periapical radiograph revealing a completely healed periapical lesion on tooth 22. **(B)** Four-year follow-up periapical radiograph demonstrating no signs of periapical pathology.

At the 3-month follow-up, the patient had no complaints, and no signs of endodontic pathology were observed. The follow-up radiograph showed periapical healing (Figure 4A). The next follow-up was scheduled for 6 months.

The patient returned after 4 years for endodontic treatment on a different tooth and reported no complaints for tooth 22. The absence of clinical signs for tooth 22 was equally followed by the complete healing of periapical tissues radiographically (Figure 4B).

## Discussion

This case report discusses the endodontic treatment of 20-year-old patient with a significant apical root resorption after orthodontic treatment and a subluxation injury to the upper left incisor. Initially managed with mineral trioxide aggregate (MTA), the tooth was later retreated using Biodentine, a new-generation hydraulic calcium silicate cement (HCSC), due to complications of secondary trauma.

Before the development of the new HCSC, MTA was the primary material recommended for vital pulp therapy, apexification, perforation, and endodontic surgery. MTA, including ProRoot MTA White used in this case, is based on ordinary Portland cement with added bismuth oxide (radiopacifier) and calcium sulfate (12). HCSC's clinical success is attributed to its ability to form calcium hydroxide during hydration, releasing calcium and hydroxide ions. Calcium activates mineralization pathways, while hydroxide ions create an alkaline environment with antibacterial and anti-inflammatory effects (13). When HCSC is applied in a target area, it may encounter an acidic environment (a pH below 7.4) due to infection and inflammation (14). However, the pH typically returns to physiological levels within 7 days after effective endodontic treatment and removal of inflamed tissues (14, 15). Additionally, HCSC can act as a buffer due to calcium hydroxide production during the setting.

Even mild dental trauma, such as subluxation, can disrupt the homeostasis of the local microenvironment due to periodontium damage and inflammatory response. In this clinical case, MTA was likely re-exposed to an acidic environment that was left initially unaddressed for several reasons: (I) there were no indications for root canal retreatment at that time; the patient was only scheduled for a regular follow-up, and (II) the alkalizing activity of calcium hydroxide is not a feature of fully set HCSC, such as ProRoot MTA, which had aged for 2 years by the time of secondary trauma. All HCSCs lack acid resistance, as the changes in physical and chemical properties have been repeatedly emphasized in the literature (14, 16). An acidic environment can initially impair the setting of HCSCs by inhibiting the formation of calcium hydroxide and calcium silicate hydrate (CSH) (14). Over time, low pH can induce the degradation of CSH, which is crucial for the strength and structural integrity (17). The decalcification of CSH begins with transformation into a lower Ca/Si ratio CSH at the pH below 8.8 and may continue until amorphous silica gel is left (18, 19). Thus, the disintegration of MTA observed at the follow-up of the secondary trauma may be due to the prolonged acidic environment in the surrounding tissues. The final explanation for the failure of the MTA may relate to the sensitivity of the material to various techniques. Factors such as inadequate condensation, moisture contamination, or premature occlusal loading could have compromised the material's matrix. However, considering the absence of symptoms for 18 months and the subsequent failure following a new trauma, environmental degradation appears to be the most likely cause.

Biodentine, which belongs to a fourth type of new-generation HCSCs and has been effectively used for endodontic retreatment following the secondary trauma in this case, could be considered a more stable and reliable option in modern endodontics (12, 20). While both Biodentine and MTA belong to HCSCs, Biodentine contains calcium chloride, accelerating the setting process and employing highly purified tricalcium silicate as a core compound. This composition results in a greater quantity of the binding calcium silicate hydrate (CSH) phase, making Biodentine less susceptible to environmental conditions than MTA (14, 20). Additionally, Biodentine is increasingly recommended for use in anterior teeth due to its lower potential for discolouration than MTA, which contains bismuth oxide.

It should also be noted that OIIRR can significantly affect the attachment of the periodontal ligament (PDL), compromising the healing response of the PDL due to an impaired reparative reaction (21). Although subluxation generally causes minimal disruption to the PDL and blood supply to the pulp, in this case, the previously orthodontically treated and OIIRR-affected tooth 22 may present a considerably diminished capacity for reparative response (7, 8). Consequently, pulp necrosis and apical periodontitis developed after the initial subluxation. Meanwhile, the compromised healing response of the PDL could explain the prolonged acidic environment in the surrounding tissues following the second subluxation, which ultimately resulted in MTA dissolution and a periapical lesion.

However, it should also be highlighted that in this particular case, the fixed wire retainer used to retain orthodontically treated teeth played a relatively favourable role (21). It acted as a passive splint, decreasing the adverse impact of secondary trauma and helping to prevent tooth displacement (7). Without the retainer, the PDL, which was already compromised due to OIIRR, could have been even more negatively affected (6). Although periapical healing was achieved with Biodentine, the reduced crown-to-root ratio and the limited length of bone-supported root suggest a cautious long-term prognosis. In a young patient, it is crucial to preserve alveolar bone, as this is vital for maintaining tooth function and ensuring that future implant options are available in case of tooth loss.

This report has limitations as it serves only as a hypothesis-generating investigation. The effects of the acidic microenvironment on material integrity were inferred from clinical context and existing literature instead of being measured directly, as this is not practical in real clinical settings. No scanning electron microscopy (SEM) or elemental analysis of the degraded MTA was performed for similar reasons. Future research should focus on measuring pH changes in teeth affected by SDT and exploring microstructural changes in the materials used.

## Conclusion

Secondary dental trauma (SDT) can compromise the quality of previous endodontic treatments, leading to material dissolution and potential reinfection. This case demonstrated that ProRoot MTA was susceptible to degradation following SDT, likely due to an altered local environment, including prolonged acidity. The retreatment with Biodentine proved to be an effective alternative, offering improved stability and biocompatibility. Additionally, orthodontically induced inflammatory root resorption (OIIRR) may further impair healing responses, increasing the risk of complications. This case report highlights the need to monitor previously traumatized teeth closely and emphasizes the importance of selecting materials resistant to environmental changes.

## Data Availability

The original contributions presented in the study are included in the article/Supplementary Material, further inquiries can be directed to the corresponding author.

## Supplementary material

Supplementary Figure S1 PRICE flowchart: an overview of the case timeline.
