# Supplementary figures and images for: Case Report: ProRoot MTA degradation in a compromised tooth: secondary trauma and acidic microenvironment leading to retreatment with Biodentine
Source: Front Dent Med. 2025 Nov 6;6:1686600. doi: 10.3389/fdmed.2025.1686600 (PMC12641284; doi:10.3389/fdmed.2025.1686600)

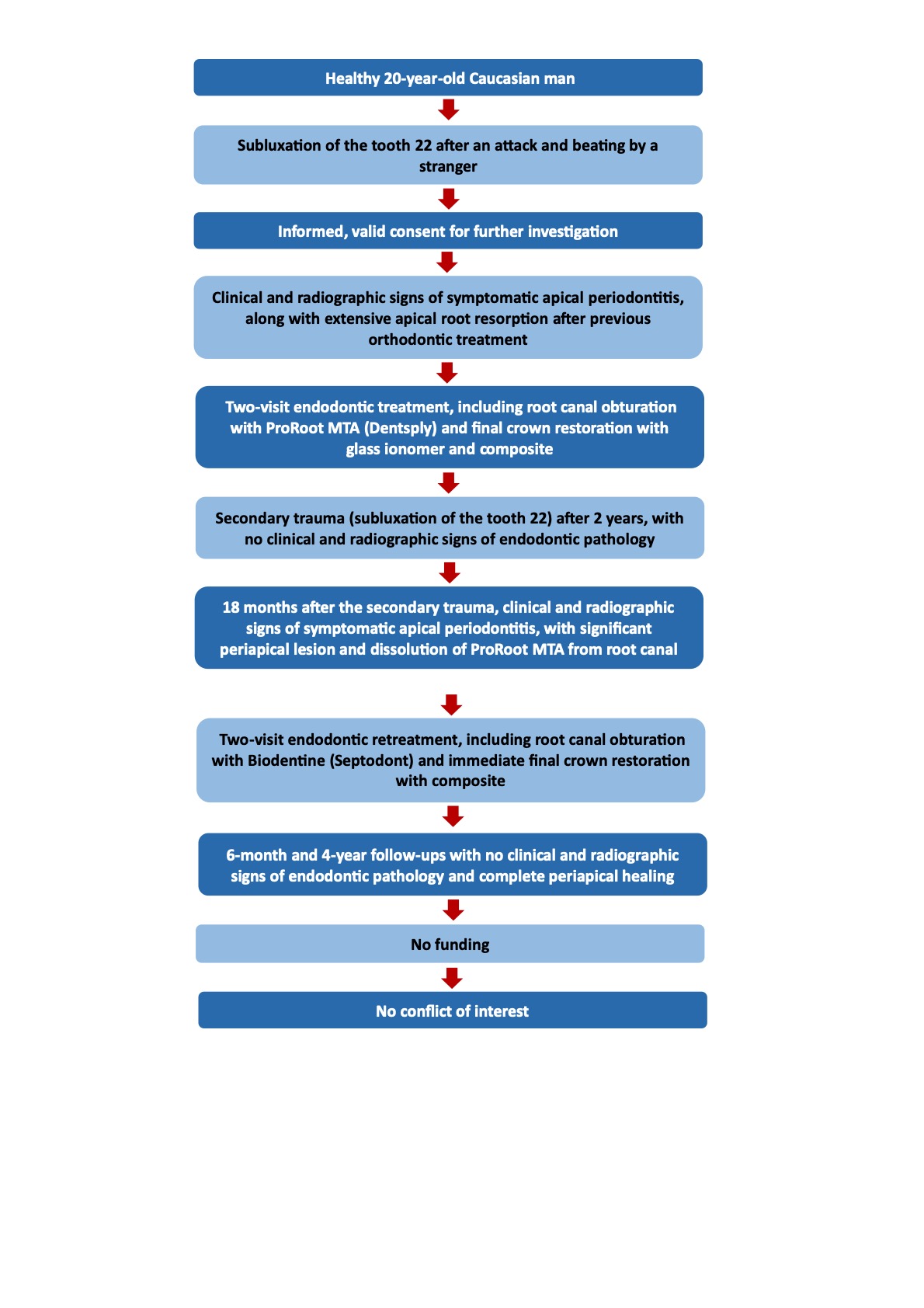

Supplement: Supplementary Figure S1 — PRICE flowchart: an overview of the case timeline. [file Image1.jpeg]
